# The Plant DNA Damage Response: Signaling Pathways Leading to Growth Inhibition and Putative Role in Response to Stress Conditions

**DOI:** 10.3389/fpls.2019.00653

**Published:** 2019-05-17

**Authors:** Maher-Un Nisa, Ying Huang, Moussa Benhamed, Cécile Raynaud

**Affiliations:** Institute of Plant Sciences Paris-Saclay, IPS2, CNRS-INRA-University of Paris Sud, Paris-Diderot and Evry, University of Paris-Saclay, Gif-sur-Yvette, France

**Keywords:** cell cycle checkpoint, DNA damage, biotic and abiotic stress, genome integrity, plants

## Abstract

Maintenance of genome integrity is a key issue for all living organisms. Cells are constantly exposed to DNA damage due to replication or transcription, cellular metabolic activities leading to the production of Reactive Oxygen Species (ROS) or even exposure to DNA damaging agents such as UV light. However, genomes remain extremely stable, thanks to the permanent repair of DNA lesions. One key mechanism contributing to genome stability is the DNA Damage Response (DDR) that activates DNA repair pathways, and in the case of proliferating cells, stops cell division until DNA repair is complete. The signaling mechanisms of the DDR are quite well conserved between organisms including in plants where they have been investigated into detail over the past 20 years. In this review we summarize the acquired knowledge and recent advances regarding the DDR control of cell cycle progression. Studying the plant DDR is particularly interesting because of their mode of development and lifestyle. Indeed, plants develop largely post-embryonically, and form new organs through the activity of meristems in which cells retain the ability to proliferate. In addition, they are sessile organisms that are permanently exposed to adverse conditions that could potentially induce DNA damage in all cell types including meristems. In the second part of the review we discuss the recent findings connecting the plant DDR to responses to biotic and abiotic stresses.

## Introduction

Maintenance of genome integrity is essential in all living organisms. It is required for proper development and for faithful transmission of the genetic information from one generation to the next. Yet, cells are constantly subjected to DNA damage. One major source of mutations is DNA metabolism itself, both during DNA replication and DNA repair. The error rate of the replication machinery is estimated in the range of 10^–7^ to 10^–8^. This low error rate results from the fidelity of replicative polymerases, which have an error rate between 10^–6^ and 10^–8^, and the successful excision of 90–99% of mis-paired bases thanks to the proof-reading activity of these complexes (Kunkel, 2004). DNA repair processes can also introduce errors, with a similar rate as replication when they involve proof-reading polymerases, or with a higher rate when they involve alternative polymerases (Kunkel, 2004; Jain et al., 2018). Finally, unrepaired lesions can block the main replicative polymerases; in that case, TransLesion Synthesis (TLS) Polymerases, take over (Uchiyama et al., 2009). They interact with each other, and are thought to form a large complex at stalled forks to allow choosing the best suited polymerase for each type of lesion (Powers and Washington, 2018). Their ability to replicate DNA passed lesions makes them error-prone: their substitution rate when replicating undamaged templates is comprised between 10^–3^ and 10^–1^ (Kunkel, 2004). In addition, DNA demethylation can also cause mutations because it requires nucleotide removal followed by Base Excision Repair (BER) (He et al., 2011).

Being sessile organisms, plants are constantly exposed to stress conditions that can also damage their DNA. Indeed, plants need light to grow photo-autotrophically, but UV light induces DNA damage, notably in the form of cyclobutane pyrimidines (CPDs). Likewise, the photosynthetic apparatus generates Reactive Oxygen Species (ROS), especially when plants are exposed to excess light, either because the intensity is very high, or when other external conditions such as heat or drought reduce the plant’s capacity to consume the reducing power produced by light absorption in photosystems (Noctor and Foyer, 2016). Very few studies have estimated the frequency of DNA lesions in plant cells. In Human cells, DNA lesions caused by spontaneous hydrolysis or ROS occur at a frequency ranging from a few hundreds to over 10^5^ per cell, depending on the type of damage (Bray and West, 2005). In maize, the number of apurinic/apyrimidic sites formed in root tips during the first 20 h of seed imbibition was estimated to 3.75 × 10^5^ per genome and per cell. Thus, although detailed quantification of DNA damage occurring in plant cells is missing, DNA damage can be considered as a frequent event under normal conditions, and likely even more so in response to various stress conditions.

In spite of the high frequency of DNA damage occurring in plant cells, the estimated mutation rate is very low. Through whole genome sequencing of Arabidopsis lines propagated from single seed descent for 25–30 generations, the genome-wide average mutation rate was estimated around 7 × 10^–9^ per site per generation (Ossowski et al., 2010; Weng et al., 2019). This figure corresponds to less than one single mutation in the entire genome per generation, and is at least 10 times lower than the error rate of the replication machinery for a single cell cycle. This provides striking evidence for the efficiency with which DNA Damage is detected and repaired in the cell. DNA lesions can be repaired through multiple pathways that have been reviewed elsewhere and will not be described into detail here (Amiard et al., 2013; Manova and Gruszka, 2015; Spampinato, 2017). Briefly, most lesions, such as UV-induced CPDs, mismatches, etc., are sensed and repaired by dedicated machineries such as photolyases, or complexes involved in mismatch repair, BER or Nucleotide Excision Repair (NER) (Jackson and Bartek, 2009; Manova and Gruszka, 2015; Spampinato, 2017). However, if incorrectly repaired, all these lesions can hamper DNA replication or cause double strand breaks (DSBs) that require specific DNA repair pathways such as Non-homologous End Joining (NHEJ) or Homologous Recombination (HR) (Amiard et al., 2013). In that case a sophisticated signaling process called the DNA Damage Response (DDR) allows activation of cell cycle checkpoints and of specific DNA repair mechanisms (Yoshiyama et al., 2013b; Hu et al., 2016). The DDR is highly conserved between eukaryotes with some variations that will be briefly discussed below. Its ultimate outcome will depend on the severity of the DNA lesions and the efficiency of the repair process: cell cycle activity can resume if lesions are successfully repaired, but more severe DNA damage can induce endoreduplication (Adachi et al., 2011). This process corresponds to several rounds of DNA replication without mitosis, leading to an increase in nuclear DNA content; it is widely distributed in plants such as in *Arabidopsis* leaves or stems, fruits, and endosperm in cereals (Galbraith et al., 1991), and is associated with cell differentiation and enlargement (Kondorosi et al., 2000). In the context of the DDR, it is thus seen as a permanent differentiation, thereby avoiding the proliferation of cells with damaged DNA. Interestingly, endoreduplication also exists in animals although it is not as common as in plants, and can be triggered by DNA damage, and could thus be a conserved response in eukaryotes (Fox and Duronio, 2013). Finally, depending on the cell type and the severity of damage, DDR activation can result in programmed cell death (PCD) (Furukawa et al., 2010). Interestingly, plant stem cells are particularly sensitive to DNA damage and prone to enter cell death (Fulcher and Sablowski, 2009), suggesting that specific mechanisms are at work to protect meristems from accumulating mutations.

The DDR signaling pathway has received extensive attention in Mammals due to its relevance in the field of cancer research, but has also been studied into details in plants for about 15–20 years. In this review we will summarize the recent advances on the plant DDR. We will focus exclusively on the DDR signaling events and cell cycle regulation, but will not discuss the complex mechanisms involved in DNA repair that have been reviewed elsewhere (Manova and Gruszka, 2015; Spampinato, 2017). Next, we will explore the emerging connection between DDR and biotic and abiotic stress responses. Indeed, even though DDR is likely activated in response to a wide range of stress conditions and could account for some of the negative effects of stress on cell division, it has to date little been studied in the context of plant response to stress, with most studies using genotoxic to trigger the DDR.

## Main Players in DDR Signaling

### ATM and ATR, the Main DNA Damage Sensors

**FIGURE 1:**
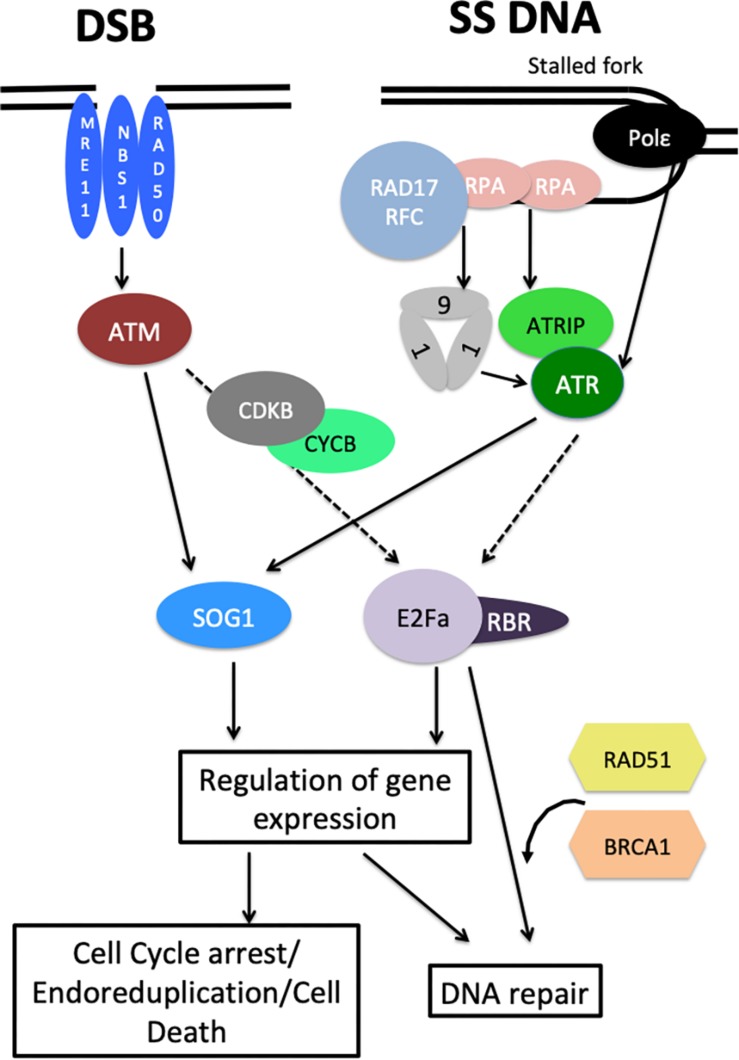
Overview of the plant DDR. DSBs activate ATM signaling through the MNR (MRE11 NBS1 RAD50) complex while ATR is recruited to single stranded DNA by RPA proteins via ATRIP, and activated by the 9-1-1 and RAD17/RFC complexes. ATR can also be activated by DNA Polymerase ε through an unknown mechanism. Both ATM and ATR signaling converge to the SOG1 transcription factor that controls the expression of hundreds of genes involved in cell cycle regulation, cell death control, and DNA repair. E2Fa/RBR complexes also control DNA repair by regulating DNA repair genes and by recruiting RAD51 and BRCA1 at DNA damage sites. The role of E2F/RBR complexes in DDR depends on CYCB1/CDKB and ATM/ATR activity, but the exact molecular mechanisms are unknown. Dashed arrows represent putative/possibly indirect regulations.

It is now well established that the general organization of the DDR signaling cascade is conserved between plants and animals. Figure 1 summarizes our current knowledge of the plant DDR. In animals, DDR activation relies on two protein kinases, called Ataxia Telangiectasia Mutated (ATM) and ATM and Rad3-related (ATR), both of which belong to the phosphatidylinositol 3-kinase-like family (Maréchal and Zou, 2013). ATM primarily responds to DSBs whereas ATR is activated by single stranded DNA and defects in replication fork progression (Maréchal and Zou, 2013); both proteins activate downstream components of the DDR. Arabidopsis homologs of ATR and ATM were isolated in the early 2000s (Garcia et al., 2003; Culligan et al., 2004), based on their sequence conservation with their counterparts in animal and yeast. Interestingly, Arabidopsis *atr* mutants are viable, in sharp contrast with *atr*-deficient mice that stop development at an early stage of embryogenesis (Culligan et al., 2004), which facilitated the functional dissection of ATR and ATM functions in plants. Like their animal homologs, ATM and ATR play both distinct and additive roles in response to DNA damage, both mutants being hypersensitive to DSBs induced by γ-irradiation whereas only *atr* is required for replicative stress response (Culligan et al., 2006). Recently, quantitative phosphoproteomics allowed the identification of hundreds of proteins that are differentially phosphorylated in response to genotoxic stress in an ATM/ATR dependent manner (Roitinger et al., 2015). This study highlighted the large number of ATM/ATR targets and thus their central role in coordinating DNA replication, DNA repair and gene expression in response to genotoxic stress.

Because they recognize different types of lesions, ATM and ATR are activated through different mechanisms. Like in animals and yeast, the plant ATM is activated by the MRN complex (MRE11, RAD50, and NBS1) that recognizes DSBs (Puizina et al., 2004; Waterworth et al., 2007; Amiard et al., 2010). In animals, ATR responds to a large variety of genotoxic stresses that all have in common to slow down DNA polymerases, leading to the accumulation of single stranded DNA. This single stranded DNA coated with the RPA (Replication Protein A) heterotrimeric recruits ATRIP (ATR Interacting Protein) which in turn facilitates the recruitment of ATR (Saldivar et al., 2017). ATR is then activated by a number of factors including the 9-1-1 complex (RAD9, RAD1, and HUS1), that is loaded on damaged DNA by the RAD17 replication Factor C 2-5 sub-units (RFC) (Saldivar et al., 2017). Furthermore in yeast, DNA Polymerase ε can directly contribute to ATR activation (García-Rodríguez et al., 2015), but whether this function is conserved in animals is unclear. The plant ATRIP protein has been identified (Sweeney et al., 2009), as well as the components of the 9-1-1 complex and RAD17 (Heitzeberg et al., 2004). It is worth noting that in plants, RPA sub-units are encoded by small multi-gene families that appear to have specialized functions in DNA replication or DDR signaling (Aklilu et al., 2014). In addition, the plant DNA Pol ε was shown to play a role in replicative stress sensing upstream of ATR, as observed in budding yeast (Pedroza-Garcia et al., 2017).

Both the ATR and the ATM pathways lead to the accumulation of γH2AX (a phosphorylated histone variant) at DNA damage sites (Amiard et al., 2010), which is instrumental for the recruitment of signaling and repair factors (Kinner et al., 2008). Intriguingly, plant *atr mre11* double mutants display a high frequency of anaphase bridges despite the complete absence of γH2AX accumulation, indicating that plants can repair DSBs in the absence of ATR and ATM activation (Amiard et al., 2010) but the underlying mechanisms remain to be fully elucidated.

### Signaling Downstream of ATM and ATR Through the Central Integrator SOG1

In animals, the ATR and ATM branches of DDR signaling converge to activate the p53 tumor suppressor, a transcription factor that controls both DNA repair and cell cycle arrest (Yoshiyama et al., 2013b). Plant genomes lack a p53 homolog, but its functional equivalent was isolated through a genetic screen for suppressors of the growth arrest induced by γ-irradiation in the *uvh1* (UV-hypersensitive 1) mutant, that is deficient for the DNA repair endonuclease XPF (Xeroderma Pigmentosum complementation group F) (Preuss and Britt, 2003). Suppressor Of Gamma-response 1 (SOG1), is a transcription factor of the NAC (NAM, ATAF1/2, and CUC2) family and is the central regulator of the plant DDR (Yoshiyama et al., 2009). It is expressed predominantly in meristems and in vascular tissues (Yoshiyama et al., 2013a), and accounts for all the short-term transcriptional changes induced by γ-irradiation (Yoshiyama et al., 2009). Genetic analysis revealed that *atm* and *atr* are partially redundant for the induction or endoreduplication or cell death in response to DNA damage, whereas SOG1 is strictly required (Furukawa et al., 2010; Adachi et al., 2011), which led to a model according to which SOG1 is the central integrator of DDR in plants (Hu et al., 2016). SOG1 is rapidly phosphorylated in response to DNA damage and is a direct target of ATM (Yoshiyama et al., 2013a) and ATR (Sjogren et al., 2015). This represents another difference between plant and animal DDR signaling, since in animals the CHK1 and CHK2 (Check point) kinases act as intermediates between ATR or ATM and p53, whereas genes encoding these kinases appear to be absent for plant genomes (Yoshiyama et al., 2013b). Recent genome-wide analyses of SOG1 targets confirmed the central role of SOG1 in the early transcriptional response to DSBs, placing SOG1 at the top of the regulatory DDR network (Bourbousse et al., 2018; Ogita et al., 2018). Surprisingly, quantitative phosphoproteomics allowed the identification of hundreds of proteins that are differentially phosphorylated in response to genotoxic stress in an ATM/ATR dependent manner (Roitinger et al., 2015) but failed to identify SOG1, possibly due to unfavorable peptide cleavage or to the fact that this study used mature rosettes while *SOG1* is mainly expressed in meristematic tissues (Yoshiyama et al., 2013a).

**FIGURE 2:**
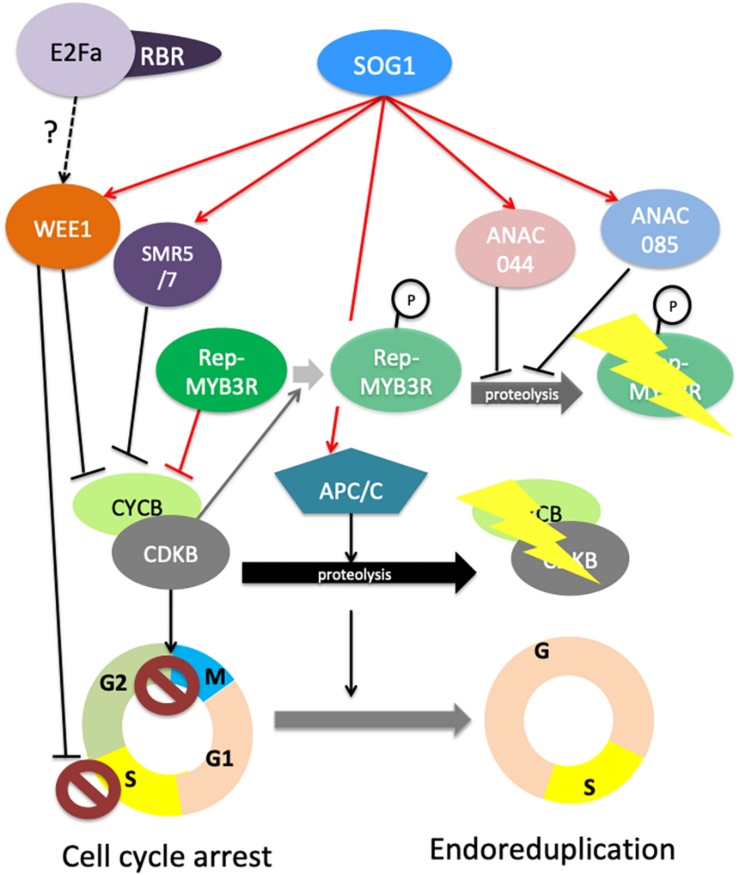
Cell Cycle regulation in response to DNA damage. Under normal conditions, protein accumulation of repressive MYB3Rs (rep-MYB3R) is restricted to S-phase during which they repress the transcription of G2 genes including *CYCBs* and *CDKBs*. Rep-MYB3R accumulation is kept low during G2 to M through phosphorylation of these transcription factors by CYCB/CDKB complexes, leading to their degradation by the proteasome (shaded shapes and arrows). Upon DNA damage, SOG1 regulates the intra S and G2/M checkpoint by targeting the core cell cycle genes *WEE1*, *SMR5* and *7* and *APC/C* sub-units, and by indirectly controlling the accumulation of rep-MYB3Rs. How SOG1 acts on MYB3Rs degradation remains to be fully elucidated, but this pathway involved direct up-regulation of the genes encoding the ANAC044 and ANAC085 transcription factors. These two proteins influence repressive MYB3R accumulation through a mechanism that remains to be elucidated, although reduction of CYCB/CDKB complexes accumulation and activity likely contributes to this process by reducing MYB3R phosphorylation. WEE1 can inhibit S-phase progression by inhibiting the activity of CYCA/CDKA complexes. WEE1 and SMR5 and 7 can also inhibit the activity of CYCB1/CDKB complexes directly, whereas MYB3R and APC/C control the accumulation of the complex. Together, all these mechanisms contribute to lowering the activity of mitotic CDKs, leading to G2 arrest or endoreduplication. In addition to these SOG1-dependent mechanisms, E2Fa/RBR complexes likely contribute to the activation of cell cycle checkpoints possibly by regulating *WEE1* or *CYC* and *CDK* genes, but their role remains to be fully elucidated. On this figure, red arrows indicate direct transcriptional regulations whereas black arrows indicate indirect regulations.

SOG1 is a transcription activator that controls the expression of DNA repair genes and cell cycle regulators (Bourbousse et al., 2018; Ogita et al., 2018). Here, we will focus on the mechanisms leading to cell cycle checkpoint activation. Depending on the phase of the cell cycle at which DNA damage occurs, cell can stop either in S phase or in G2. Replicative stress activates an intra-S checkpoint that is dependent on SOG1 and WEE1 (De Schutter et al., 2007; Cools et al., 2011; Hu et al., 2015), a protein kinase that stops the cell cycle through an inhibitory phosphorylation of Cyclin Dependent Kinases (CDK). SOG1 can also induce a G2 arrest of the cell cycle through several mechanisms. First, together with ATR, SOG1 was shown to control proteasome-dependent degradation of the mitotic CDKB2;1 (Adachi et al., 2011); second, SOG1 controls the expression of genes encoding negative cell cycle regulators such as the CDK inhibitors SMR5 and SMR7 that induce endoreduplication (Yi et al., 2014). Furthermore, the WEE1 kinase inhibits CDK activity (De Schutter et al., 2007; Cools et al., 2011; Cook et al., 2013), thereby inhibiting the G2/M transition, and SOG1 also stimulates the expression of the G2-specific CYCLINB1, a mechanism that has been proposed to delay mitosis, although it likely also reflects the specific involvement of CYCB1;1 in DNA repair (Schnittger and De Veylder, 2018). More recently, the full analysis of SOG1-dependent transcriptome changes induced by DNA damage, further revealed that SOG1 partly acts through the activation of MYB3R repressors that inhibit the expression of G2/M cell cycle genes (Bourbousse et al., 2018). MYB3R transcription factors are well known regulators of the G2/M transition, MYB3R4 being an activator, MYB3R3 and 5 repressors, and MYB3R1 behaving either as an activator or as a repressor depending on its interacting partners (Haga et al., 2011; Kobayashi et al., 2015a, b). Recently, Chen et al. (2017) demonstrated that repressor MYB3Rs (Rep-MYB3R) are essential for the growth inhibition induced by DNA damage: in response to zeocin treatment, the MYB3R3 protein accumulates in root meristems, thereby preventing cell proliferation by inducing a G2 arrest. In this work, authors showed that MYB3R3 is phosphorylated by CDKs and that this phosphorylation promotes its proteasomal degradation. Thus, reduction of CDK activity due to CDK inhibitors induction likely contributes to the accumulation of Rep-MYB3Rs in response to DNA damage. Together, these observations shed new light on the mechanisms underlying the SOG1-dependant repression of CDKB2;1 accumulation. Indeed, SOG1 positively regulates activators of the Anaphase Promoting Complex/Cyclosome (APC/C) (Bourbousse et al., 2018). The down-regulation of CDKB2;1 in response to DNA damage could thus result from the concomitant degradation of the protein by the APC/C and repression of the CDKB2;1 gene by Rep-MYB3Rs. Very recently, the ANAC044 and ANAC085 transcription factors, the two SOG1 closest relatives that are also SOG1 targets (Ogita et al., 2018), were reported to promote rep-MYB3R accumulation in response to DNA damage (Takahashi et al., 2019). Genetic analysis showed that ANAC044 and ANAC085 function in the same pathway as SOG1 to control cell cycle arrest through rep-MYB3R accumulation but not activation of *SMR* genes or DNA repair genes. To date, it remains unclear how ANAC044 and ANAC085 modulate Rep-MYB3R protein levels, as they do not directly target *Rep-MYB* genes, but this pathway could involve the regulation of proteins involved in the degradation of Rep-MYBs such as F-box proteins (Takahashi et al., 2019). Figure 2 summarizes how DDR triggers cell cycle arrest either in S phase or in G2 phase, and can lead to cell differentiation and endoreduplication.

### EF2/RBR Complexes: New Players in the Plant DDR

Despite this central role of SOG1, recent studies have revealed SOG1-independent pathways in the plant DDR. The first evidence for SOG1-independent DDR response came from the genetic analysis of *wee1 sog1* double mutants, that showed enhanced sensitivity to replicative stress compared to the *sog1* mutant, providing evidence for a SOG1-independent mechanism that could lead to WEE1 activation (Hu et al., 2015). This hypothesis is further supported by the analysis of Arabidopsis mutants with partial deficiency in the replicative DNA Polymerase ε that suffer from constitutive replicative stress (Pedroza-Garcia et al., 2016, 2017). ATR and WEE1 are both essential for the survival of *abo4-1* mutants that are partially deficient for the Pol ε catalytic subunit, whereas the *abo4-1 sog1* double mutants are viable. Consistently, some DDR responsive genes are induced in a SOG1-independent manner in the *abo4-1 sog1* double mutants (Pedroza-Garcia et al., 2017). The underlying molecular mechanism remains unknown, but may involve E2F-RBR1 (RetinoBlastoma Related 1) complexes. These transcription regulators are well known both in plants and animals for controlling S-phase entry: RBR1 binds and inhibits E2F transcription factors thereby preventing the expression of S-phase genes (Berckmans and De Veylder, 2009). Upon activation of CYCD-CDKA complexes and cell cycle entry, RBR1 is phosphorylated and E2F transcription factors function together with their Dimerization Partners (DP) proteins to activate the expression of genes involved in DNA replication, leading to the onset of S-phase (Gutzat et al., 2012). Besides its role in cell cycle regulation, E2Fa had been previously shown to control the expression of RNR (RiboNucleotide Reductase), an enzyme involved in deoxyribonucleotide biosynthesis that is strongly activated by DNA damage (Roa et al., 2009). Furthermore, E2Fa was shown to form foci at DNA damage sites (Lang et al., 2012). Two recent studies further substantiated the role of RBR1 and E2Fs in the plant DDR: a temperature sensitive *rbr1* mutant was shown to be hypersensitive to DNA damage, and to accumulate enhanced levels of DNA lesions in response to genotoxic stress (Biedermann et al., 2017), while *RBR1* silencing triggered DNA damage accumulation and cell death onset in root tips even in the absence of exogenous stress (Horvath et al., 2017). Intriguingly, RBR1 represses the expression of several DDR genes in a E2Fa-dependent manner (Biedermann et al., 2017; Horvath et al., 2017), and RBR1 deficiency could thus have been expected to improve the DNA repair capacity of the plant. However, authors also demonstrated that RBR1 localizes to DNA damage foci (Biedermann et al., 2017) together with E2Fa, and recruits the DNA repair proteins RAD51 (RADIATION SENSITIVE 51) and BRCA1 to the DNA damage site (Biedermann et al., 2017; Horvath et al., 2017). Thus E2F-RBR1 could play a dual role in the DDR (i) by controlling the expression of DDR genes, possibly to up-regulate their expression during S-phase and thereby enhance the repair activity at this specific phase of the cell cycle that triggers extensive DNA damage, and (ii) more directly by controlling the DNA repair process itself at specific sites (Figure 1). Moreover E2F/RBR complexes contribute to cell cycle checkpoint activation during DDR: loss of RBR results in enhanced cell death in response to genotoxic stress, suggesting that E2F/RBR complexes function antagonistically to SOG1 to restrict PCD (Biedermann et al., 2017). Further, since ATR and WEE1, but not SOG1 are required for the survival of Pol ε deficient mutants that display constitutive replicative stress, RBR/E2F complexes may play a role in the control of the intra-S checkpoint, possibly by controlling *WEE1* or *CDK/CYC* expression (Figure 2). In line with this hypothesis, RBR was found to target *WEE1* and a large number of core cell cycle regulators as well as many DNA repair genes (Bouyer et al., 2018). How E2F-RBR complexes are regulated upon DNA damage remains to be fully clarified. Formation of RBR foci upon DNA damaged was reported to depend both on CYCB1/CDKB and ATM/ATR activity (Biedermann et al., 2017; Horvath et al., 2017). Whether RBR is directly phosphorylated by ATM, ATR and CYCB1/CDBK1 complexes, or whether the kinases function sequentially remains to be established. Neither RBR nor CYCB1/CDKB have been identified as putative ATM/ATR targets (Roitinger et al., 2015). Further work will thus be needed to fully dissect this part of the DDR signaling cascade.

Besides RBR1, another regulator called SNI1 (Suppressor of Npr1 Inducible 1) was recently reported to antagonize E2Fs, and was proposed to have a dual function in the DDR by connecting cell cycle checkpoint activation and DNA repair mechanisms (Wang et al., 2018). SNI1 is a subunit of SMC5/6 complex (Structural Maintenance of Chromosome), which is conserved in all eukaryotes (De Piccoli et al., 2009). Over-expression of *SNI1* rescues the phenotype of E2Fa/DPa over-expressers that is characterized by increased endoreduplication level and retarded growth (De Veylder et al., 2002), likely because it represses E2F target genes through the recruitment of histone deacetylases (Wang et al., 2018). Reciprocally, loss of E2Fs abolishes the induction of cell death observed in the root tip of *sni1* mutants. Interestingly, loss of genes involved in HR had been previously reported to suppress cell death in *sni1* mutants (Durrant et al., 2007; Wang et al., 2010; Song et al., 2011). Since RBR1 and E2F are recruited to a small number of foci associated with heterochromatin, and not to all DNA damage sites, it is thus tempting to speculate that RBR1/E2F complexes and SNI function in heterochromatin-specific DNA repair mechanisms. Indeed, in human cells, the choice between DSB repairs pathway is greatly influenced by chromatin compaction, heterochromatin being more prone to Non-homologuous End Joining (NHEJ) possibly to avoid HR between repeats (Lemaître and Soutoglou, 2014).

All the above-mentioned studies have been conducted in Arabidopsis, using DNA damaging agents. However, understanding and characterizing the contribution of plant DDR pathways in more physiological conditions could provide valuable insight into the plant response to various environmental stresses.

## Role of the Plant DDR in Abiotic Stress Responses

Although studies connecting the plant DDR to abiotic stress responses remain scarce, maintenance of genome integrity is likely to play a role in plant stress tolerance. In agreement with this hypothesis, whole genome sequencing of two species of *Eutrema*, a recently evolved genus of alpine Brassicaceae, revealed that several genes involved in DNA repair, cell cycle regulation or DDR are duplicated, thereby providing a potential mechanistic basis for the adaptation of these plants to the harsh alpine environment (Guo et al., 2018). Indeed, a number of abiotic stresses are well known to cause DNA damage. The most obvious example is UV-B light (280–320 nm) that directly damages DNA by inducing the formation of CPDs. This results in DNA strand distortion, and hampers both transcription and DNA replication (Britt, 2004). Most CPDs are directly repaired by photolyases such as UVR2 (UV Response 2) in Arabidopsis (Willing et al., 2016), but tolerance of UV-B photodimers also requires TLS polymerases to allow DNA replication to proceed in spite of lesions (Curtis and Hays, 2007). When unrepaired, CPDs can activate the DDR. Indeed, exposure to UV-B light, like γ-irradiation, can induce PCD in root meristems, in a SOG1-dependent manner (Furukawa et al., 2010). PCD induction after γ-irradiation still occurs in *atm* and *atr* single mutants, although it is delayed, but not in double mutants, indicating that either kinase is sufficient to activate SOG1 (Furukawa et al., 2010). Likewise, zeocin-induced cell death was abolished in both *atm* and *atr* mutants in the root tip, while it seems to require only ATM in the inflorescence meristem, suggesting that DDR signaling components play partially specialized functions depending on cell types (Fulcher and Sablowski, 2009). In maize and Arabidopsis, histone acetylation has been associated to UV-B responses and damage repair (Campi et al., 2012; Fina et al., 2017). Interestingly, mutants deficient for histone acetyltransferases showed reduced growth inhibition after UV-B exposure, associated with altered expression of E2F transcription factors (Fina et al., 2017). Consistently, E2Fc know-down lines show less severe reduction of leaf growth in response to UV-B than the wild-type, suggesting that E2Fc could also play a role in the DDR activated by UV light (Gómez et al., 2019), as was previously suggested for the atypical E2Fe (Radziejwoski et al., 2011).

Another well documented example of abiotic stress activating the plant DDR is the exposure to heavy metals [for example cadmium (Cd), copper (Cu), lead (Pb) or mercury (Hg) (Küpper and Andresen, 2016; Lanier et al., 2019)], or other metallic ions such as aluminum (Al). These metallic ions can be divided into two categories: some, like copper or zinc are essential for plant growth but toxic at high doses, while others such as cadmium, mercury, and lead are not required for plant development. The toxic effects of these metals are varied, ranging from impairment of photosynthesis to inhibition of the uptake of other essential metal ions, but many of them cause DNA damage either directly, or through the induction of ROS production (Küpper and Andresen, 2016).

Among the metal elements that can affect plant growth, Al is probably one of the best studied, because it is very abundant, and because Al^3+^ ions that are predominant in acidic soils cause severe phytotoxicity, making this metal one of the primary growth limiting factors for agriculture. Exposure to Al^3+^ was shown to induce DNA damage in Arabidopsis (Nezames et al., 2012; Chen et al., 2019) but also in crops such as barley (Jaskowiak et al., 2018), and plant growth inhibition in response to this ion has been shown to require ATR and SOG1 (Rounds and Larsen, 2008; Sjogren et al., 2015; Zhang et al., 2018). Since Al causes DSBs, the improved root growth of *sog1* or *atr* mutants on Al containing medium may appear counter-intuitive. However, detailed genetic dissection of the response to low and high doses of Al allowed Chen and colleagues to propose a model according to which low levels of Al-induced DNA damage triggers ATR-dependent SOG1 activation leading to growth reduction and CYCB1/CDKB-dependent DNA repair. This pathway can be inactivated without compromising plant survival, suggesting that another pathway can allow activation of CYCB1-dependent DNA repair in the absence of ATR and SOG1. This alternative activation mechanisms could rely on RBR1 since *rbr1* mutants are hypersensitive to Al (Biedermann et al., 2017). By contrast, response to higher doses of Al and more severe DNA damage involves ATM-dependent SOG1 activation triggering the full activation of the DDR and leading to minimal growth, this pathway being indispensable for plant survival (Chen et al., 2019).

In addition to the well documented examples of UV-light or metal ions, there is accumulating evidence that a wide variety of stress conditions can induce DNA damage through unknown mechanisms that could involve ROS production. For example, prolonged chilling stress was found to induce DNA fragmentation in tobacco BY-2 cells (Koukalova et al., 1997) or maize root tip cells (Ning et al., 2002). Although one cannot rule out that some of the DNA damage observed in plants after exposure to stress is a consequence of the onset of PCD rather than actual stress-induced DNA damage, cold stress has been shown to increase oxidative DNA damage in roots of *Cardamine pratensis* (Białkowski and Oliński, 1999). Consistently, Hong et al. (2017) recently reported that DDR activation in the root tip was essential to meristem survival after chilling stress. According to their model, cold stress induces DNA damage in the root tip, leading to selective PCD onset in the columella stem cell daughters. This response requires the canonical DDR players ATM, ATR, SOG1, and WEE1 and allows maintenance of the local auxin maximum in the root tip, thereby protecting meristem organization and allowing recovery after stress (Hong et al., 2017). Whether similar processes are activated in response to other kinds of stresses such as excess light, heat or drought remains to be fully explored, but a few studies support this notion. Indeed, the ANAC044 and ANAC085 transcription factors were found to promote cell cycle arrest in response to heat stress. Although this response is independent of SOG1, this finding demonstrates that some DDR components can be recruited in response to other types of abiotic stresses to induce cell cycle arrest (Takahashi et al., 2019). Furthermore, ozone induces DNA damage in wheat, particularly under water limiting conditions and heat or high light severely enhance DNA damage accumulation in rice mutants deficient for RNase H2 (Qiu et al., 2019). Activation of DNA repair also likely plays a key role during dehydration and rehydration in resurrection plant (Liu et al., 2018). Consistently, expression of a number of cell cycle inhibitors is induced in response to abiotic stresses, and *SMR5* and *7*, that are direct SOG1 targets have been shown to promote early exit of the cell cycle in response to chloroplastic stress (Hudik et al., 2014). Interestingly, *SMR5* is induced in response to heat, drought or high-light (Yi et al., 2014), and the same study revealed that SOG1 is phosphorylated in response to H_2_O_2_ accumulation, suggesting that generally, stress-induced ROS accumulation could trigger DDR activation. In agreement with this hypothesis, loss of the ROS detoxifying enzymes Ascorbate Peroxidase and Catalase 2 results in the activation of a WEE1-dependent cell cycle checkpoint (Vanderauwera et al., 2011) resulting in growth inhibition. Although this study was conducted in mutants in which ROS detoxification is severely compromised, it suggests that a similar response could be activated in wild-type plants exposed to stress. Together, these observations support the notion that many, if not all abiotic stresses, can activate the DDR, which could contribute to the plant growth reduction that is a common for all stress responses (Claeys et al., 2013). In this context, a better understanding of the plant DDR would open possible opportunities to counter environmentally induced yield-loss.

Finally, the plant DDR is clearly instrumental for seed viability and seedling vigor (Ventura et al., 2012; Waterworth et al., 2015). Indeed, both seed dehydration and germination, which are accompanied by a burst of ROS production, are highly damaging for DNA, and up-regulation of DNA repair genes during germination is well documented in Arabidopsis (Waterworth et al., 2010), *Medicago truncatula* (Balestrazzi et al., 2011) and *Phaseolus vulgaris* (Parreira et al., 2018). Consistently, *atm* mutants fail to delay germination in aged seeds, and show extensive chromosomal abnormalities (Waterworth et al., 2016), and HR-deficient or DDR mutants are hypersensitive to ABA during germination and at the seedling stage (Roy and Das, 2017). Thus, the probable contribution of the plant DDR to abiotic stress tolerance is supported by its essential role during germination, a particularly stressful step of the plant life cycle.

Maintenance of genome integrity is well known to be essential for meristem function, as illustrated by numerous examples of mutants affected in DNA Damage repair in which meristem organization is perturbed or its function is lost [e.g., (Wenig et al., 2013; Li et al., 2017; Han et al., 2018)], and it would thus not be surprising to find that DDR activation is a key factor for plant survival under abiotic stress conditions. In line with this hypothesis, DDR has been shown to shape directly or indirectly plant development in response to stress. In the root meristem, replacement of damaged stem cells relies on the reactivation of the ERF115 transcription factor to promote cell division (Heyman et al., 2013), and its transcriptional up-regulation occurs in cells that are in direct contact with damaged cells (Heyman et al., 2016). In the context of DNA-damage induced PCD, *ERF115* induction was shown to depend partially on SOG1 activity (Johnson et al., 2018). DDR was also shown to impact lateral root formation by modulating cytokinin signaling (Davis et al., 2016), and to account for the reduction of hypocotyl growth triggered by UV (Biever et al., 2014), suggesting that its activation could contribute to the well-known plasticity of plant development according to external conditions.

## Role of the Plant DDR in Biotic Stress Response

A similar connection can be drawn between the plant DDR and response to biotic stresses. It has long been known that pathogen infection or treatment with the defense hormone Salicylic Acid (SA) stimulates HR, suggesting that the DDR is activated by biotic stress (Lucht et al., 2002; Kovalchuk et al., 2003). Consistently, Song and colleagues reported that a variety of pathogenic, and even non-pathogenic micro-organism induce DNA damage in plant cells (Song and Bent, 2014). However, this accumulation of DNA damage does not depend on pathogen-induced ROS production, and the underlying mechanisms thus remain unknown. SA treatment has been shown to induce DNA damage (Yan et al., 2013) but this effect is debated, since in another study pre-treatment with SA was found to reduce DNA damage accumulation in response to infection, and SA alone failed to induce DNA damage (Song and Bent, 2014).

Thus the mechanisms leading to DNA damage accumulation during infection remain unclear, although some of these DNA lesions could simply reflect the induction of PCD as a defense mechanism. Nevertheless, there is accumulating evidence that DDR activation is relevant to plant immunity. First a number of DNA repair mutants have been reported to show enhanced susceptibility to *Pseudomonas syringae*. This is the case for plants lacking PARP2 (Poly ADP-ribose polymerase) that plays an important role for DNA repair (Song et al., 2015), and mutants affected in DSB repair by HR such as *rad51* or *brca2* (Durrant et al., 2007; Wang et al., 2010; Song et al., 2011). Second, DDR signaling mutants such as *atm*, *atr*, or *rad17* have also been reported to be more susceptible to *P. syringae* (Yan et al., 2013; Song and Bent, 2014). One possible explanation for these observations would be that an efficient DDR activation and DNA repair is required for plant cell survival in response to biotic stress, possibly to avoid cell death due to the accumulation of DNA lesions; or on the contrary to contribute to PCD induction to limit pathogen growth. However, there is evidence that the DDR could enhance plant defense activation. Indeed, the above-mentioned *SNI1* gene was initially isolated as a negative regulator of systemic acquired resistance in a suppressor screen of the *npr1* (Non-expressor of PR genes) mutant (Li et al., 1999). As previously stated, *SNI1* was found to encode a sub-unit of the SMC5/6 complex (Yan et al., 2013) that plays a crucial role in DNA repair, notably in the removal of post-replicative damage (Diaz and Pecinka, 2018). *Sni1* mutants constitutively accumulate DNA damage, and show enhanced tolerance to pathogens, suggesting that DDR activation could stimulate biotic stress responses (Yan et al., 2013). Consistently, the recent genome-wide identification of SOG1 target genes revealed that a number of defense-related genes are SOG1 targets, providing a direct link between biotic stress and DDR (Bourbousse et al., 2018; Ogita et al., 2018).

In addition, DNA repair proteins have been proposed to play a direct role in the control of immune responses: activation of defense-related genes by SA in the *npr1 sni1* double mutant was largely dependent on BRCA2 (Wang et al., 2010). Furthermore, RAD51 and BRCA2 appear to directly bind the promoter of the *PR1* and *PR2* defense genes (Wang et al., 2010). These results thus led to a model according to which BRCA2 and RAD51 would directly control the transcription of immunity-related genes. However, the primary defect of the *sni1* mutant likely is in DNA repair since *sni1* is a sub-unit of the SMC5/6 complex (Yan et al., 2013). The accumulation of DNA damage in the *sni1* mutant is largely alleviated in the *atr* background (Yan et al., 2013), suggesting that the DNA lesions accumulate because ATR signaling triggers repair mechanisms that cannot be fully completed, possibly due to the absence of SMC5/6. In the absence of ATR activation, alternative pathways must be activated leading to a reduction of DNA damage accumulation. Under such a scenario, the activation of defense genes in *sni1* could be an indirect effect of DDR activation, possibly through the activation of SOG1. In that case, one could hypothesize that BRCA2 and other DNA repair proteins could contribute to the accumulation of repair intermediates that trigger the DDR. Loss of these proteins, including ATR, could reduce DNA damage accumulation by allowing alternative repair mechanisms to function, and thus DDR signaling through SOG1. In line with this hypothesis, 163 out of the 265 BRCA2-dependent defense genes identified by Wang et al. (2010) are differentially expressed in response to γ-irradiation according to Bourbousse et al. (2018). Thus, to fully ascertain the direct role of BRCA2/RAD51 complexes in immunity, the effect of the *brca2* or *rad51* mutations on defense gene expression should be analyzed in a wild-type background and a genome-wide analysis of BRCA2/RAD51 target genes during biotic stress response should be performed. Whether DNA repair proteins directly control the expression of several defense genes or not, there is converging evidence for a role of the plant DDR during immunity, which could, as we proposed in the case of abiotic stress, contribute to the growth inhibition induced by pathogens.

## Concluding Remarks

The plant DDR is emerging as a key process shaping plant growth and development in response to environmental cues. Now that the main actors of this signaling pathway have been characterized, future work should elucidate the molecular connections between DDR and plant response to stress, thereby opening new prospects for crop improvement. Another promising line of research will be to decipher the connections between the DDR and chromatin dynamics. Indeed, replicative stress has been shown to affect the maintenance of gene silencing through DNA replication in yeast (Sarkies et al., 2010), a mechanism that most likely applies to plants, as evidenced by the large number of DNA replication proteins isolated in genetic screens for suppressors of silencing (Kapoor et al., 2005; Yin et al., 2009; Liu et al., 2010; Hyun et al., 2013). Furthermore, DNA repair processes require extensive chromatin remodeling to allow access of the repair machinery to DNA (Nair et al., 2017). Thus DNA damage represents a challenge for chromatin maintenance. Reciprocally, defects in chromatin dynamics can lead to genome instability and DNA damage accumulation (Ma et al., 2018). Mechanisms allowing chromatin reconstruction after DNA repair or connecting chromatin dynamics with genome stability have been little explored, particularly in plants, and will likely receive increasing attention in the future.

## Author Contributions

M-UN wrote the paragraph on DDR signaling. YH wrote the paragraph on DDR and abiotic stress. MB and CR conceived the review organization and figures. CR wrote the rest of the text and supervised the writing.

## Conflict of Interest Statement

The authors declare that the research was conducted in the absence of any commercial or financial relationships that could be construed as a potential conflict of interest.
